# Safety of equine tetanus antitoxin for prophylactic use in Ethiopia: a retrospective multi-center study

**DOI:** 10.1186/s41182-023-00518-8

**Published:** 2023-05-05

**Authors:** Michele Joseph, Yimtubeznash Woldeamanuel, Girmay Medhin, Tsegahun Manyazewal, Abebaw Fekadu, Eyasu Makonnen

**Affiliations:** 1 Center for Innovative Drug Development and Therapeutic Trials for Africa (CDT-Africa), College of Health Sciences, Addis Ababa University, P.O. Box 9086, Addis Ababa, Ethiopia; 2 Department of Microbiology, Immunology and Parasitology, College of Health Sciences, Addis Ababa University, Addis Ababa, Ethiopia; 3 Aklilu Lemma Institute of Pathobiology, Addis Ababa University, Addis Ababa, Ethiopia; 4 Department of Psychiatry, College of Health Sciences, Addis Ababa University, Addis Ababa, Ethiopia; 5 Department of Pharmacology and Clinical Pharmacy, College of Health Sciences, Addis Ababa University, Addis Ababa, Ethiopia

**Keywords:** Tetanus, *Clostridium tetani*, Antitoxin, Equine, Safety, Adverse events following immunization (AEFI), Ethiopia

## Abstract

**Background:**

Tetanus remains a severe life-threatening infectious disease and neurological disorder in many parts of the world, where immunization programs are suboptimal. Any human injury or trauma has the possibility of getting infected with *Clostridium tetani* which is the sole causative bacterium of tetanus. Evidence is available that TAT may cause anaphylaxis and late serum sickness, while there has been no study conducted in Ethiopia. The Ethiopian Ministry of Health standard treatment guideline recommends tetanus prophylaxis for all tetanus-prone wounds. This study aimed to evaluate the safety of TAT administration in adults exposed to tetanus-prone wounds in Ethiopia.

**Methods:**

The target product of this study was the equine tetanus antitoxin developed and manufactured by the ViNS Bioproducts Limited, India (Code: 130202084, A.W.No: 15/AAW/PI/02.00, DT: 25.04.2016). The product is delivered with the dose of 1000/1500 IU intramuscularly or subcutaneously to individuals at risk of tetanus infection for prophylactic purposes. The study was carried out in 11 healthcare facilities in Addis Ababa, Ethiopia, that had a relatively high clients load for tetanus-prone wounds. Medical records of patients with tetanus-prone wounds who received the equine TAT were reviewed retrospectively for any adverse events following immunization according to the World Health Organization (WHO) definition for adverse events following immunization (AEFI).

**Results:**

There were more than 20,000 patients treated for trauma in the facilities from 2015 to 2019. Upon revision of available registration books, we identified 6000 charts to be eligible for the study, of which 1213 charts that had complete and reliable data on the AEFI profile of the TAT were included in the final analysis. The median age of the study participants was 26 years (IQR = 11 years, age range: 18–91 years) and 78% (949) were male. The tetanus-prone wounds resulted mainly from stab (44%, 535) and blunt force (30%, 362), and the most common sites of wounds were hand (22%, 270) and head (21%, 253). The most and least frequently occurring types of wounds were open wounds (77%, 930) and organ system injury (0.003%, 4), respectively. The mean time of presenting at health facilities from the onset of trauma was 2.96 h. Of the total 1231 participants, one male participant who presented within 3 h after experiencing a wound on his nose at the workplace had a severe local reaction immediately after injection of the TAT. No AEFI was recorded for the other participants.

**Conclusions:**

The adverse event following immunization of the equine tetanus antitoxin produced by the ViNS Bioproducts Limited was very rare. A regular review of the product’s safety performance and systematic collection and analysis of adverse event reports are important to ensure the safety of the product.

## Introduction

Tetanus is a bacterial infection that affects the central nervous system. The causative agent, *Clostridium tetani*, is a spore-forming, toxin-producing gram-positive, anaerobic bacillus found in the environment irrespective of geographical location [1, 2]. The spores are extremely resistant to heat and conventional antiseptics, being able to survive for many years in adverse conditions. Tetanus may follow puncture wounds, surgery, burns, crush wounds, otitis media, animal bites, oral infections, or childbirth and abortion. The spores enter the body through these routes and produce the toxins tetanolysin and tetanospasmin in anaerobic conditions [3, 4]. The toxin migrates to the central nervous system through retrograde axonal transport within nerve cells and causes muscular rigidity and spasms [5]. The incubation period is usually 8–10 days (range 3–21 days) following infection of a wound. Among patients in the youngest and oldest age groups, the case-fatality rate approaches 100% without intensive care [6]. Diagnosis is entirely based on clinical features and does not depend on laboratory confirmation, for *C. tetani* is recovered from the microbiologic culture of wounds in only about 30% of cases [6, 7]. The combination of a history of injury with a tetanus-prone wound and muscular spasms could increase the possibility of the infection [8]. Wound management, a single intramuscular dose of human or equine tetanus antitoxin, and antibiotics prevent further progression of the disease [7].

Epidemiologically, tetanus remains an important public health problem in many parts of the world, where immunization programs are suboptimal. It causes an estimated 56,000 deaths annually worldwide, approximately 20,000 of them are neonates [3], while in many countries, tetanus disease surveillance is not well-established, and thus its incidence is not accurately known. Among global tetanus deaths, 44% occur in sub-Saharan Africa and the highest proportion of these is in East Africa [9]. In Africa, there were 2791 and 5787 reported cases of Tetanus in 2017 and 2018, respectively [10]. In Ethiopia, there were 20 reported cases of tetanus in 2017 as per the WHO report [10].

Prophylaxis of tetanus consists of local wound management, active immunization, and passive immunization. An active immunization, a six-dose series of tetanus toxoid vaccine, protects by stimulating the production of antitoxin, providing immunity against the effects of the toxin [3]. A passive immunization, which could be tetanus immunoglobulin (TIG, human origin) or tetanus antitoxin (TAT, equine origin), is recommended as post-exposure-prophylaxis within 24 h after a tetanus prone wound has occurred. Both TIG and TAT are currently available in the global market. The antitoxins neutralize any circulating toxin before it reaches the nervous system [11], but cannot remove the toxin that is bound to nerve endings [8]. Due to the strong effect of its toxins, tetanus infection does not produce tetanus immunity [8].

Equine tetanus antitoxin (TAT) is made of toxin-neutralizing immunoglobulin fragments F (ab’)2, extracted and purified from the serum of tetanus toxoid-immunized horses [12]. In persons who have not had horse serum previously, the antitoxin reaches very high levels in the serum during the first week after injection, but the human host treats it as a foreign protein and its level drops sharply after the 10th day [13].

It has been estimated that 5–6% of adult patients receiving TAT will have some adverse reaction [14], while not contraindicated in case of pregnancy or breastfeeding [13]. Anaphylactic shock may occur despite a negative sensitivity test or may result from the test dose itself [15]. It has a risk of hypersensitivity and serum sickness. It should not be administered to patients with a known allergy to tetanus antiserum. It may cause hypersensitivity reactions, anaphylactic shock, Quincke edema, or serum sickness up to 10 days after injection [13].

Human TIG was developed in 1960 due to the side effects of the equine tetanus antitoxin [11]. Human TIG is more efficacious and long-lasting than TAT [16]. In most developed countries, TAT is totally replaced by TIG, while TAT products are still in use in developing countries in view of the high costs and limited access to human TIG [17]. Though TIG is available in Ethiopia, it is not available as much as TAT due to its cost, forcing the health care system to use the alternative TAT as post exposure prophylaxis.

In Ethiopia, recent studies have reported a high case-fatality rate of patients admitted to hospitals due to tetanus infection [18–20] and a low history of tetanus toxoid vaccination [20–22]. The healthcare provider needs to determine the patient's tetanus immune status; if the status is inadequate or unknown, the provider should administer Human TIG or TAT with close follow-up of the potential adverse events following immunization.

For equine driven TAT, though there have been no formal studies conducted in Ethiopia, evidence is available that they can cause anaphylaxis [23], late serum sickness [23, 24], while these products are still in use due to their low costs [25, 26]. Immediate systemic allergic reactions after administration of this product have not been documented in Ethiopia.

Thus, conducting a retrospective safety study on the TAT product manufactured by ViNS Bioproducts Ltd is important to evaluate the safety of equine tetanus antitoxin, promote public health, and to support the improvement of the product by providing scientific evidence from a local context.

This study aimed to evaluate the safety of equine tetanus antitoxin, (Code: 130202084, A.W.No: 15/AAW/PI/02.00, DT: 25.04.2016, VINS Bioproducts Limited, India) when administered to adults under conditions of routine post-exposure prophylactic use in Ethiopia.

## Methods

### Study design and material

This was a multi-site retrospective cohort study on safety of TAT for prophylactic use. The study was carried out in 11 public healthcare facilities (three referral hospitals and eight primary health centers) in Addis Ababa, Ethiopia, that had a relatively high clients load for tetanus-prone wounds. Reports at the Addis Ababa Health Bureau and the Ethiopian Pharmaceutical Supply Agency were reviewed to identify and select the sites with high TAT clients load.

The subject of this study is equine tetanus antitoxin which is developed and manufactured by the ViNS Bioproducts Limited, India [27] (Code: 130202084, A.W.No: 15/AAW/PI/02.00, DT: 25.04.2016). The product has been licensed and in use in Ethiopia since 2015. It is prepared by hyperimmunized horses with tetanus toxoid. Plasma obtained from healthy immunized horses is enzyme refined, purified and concentrated. The tetanus antitoxin has specific antitoxic immunoglobulins which neutralize the toxin formed by *C tetani*. The product is supplied as 1 ml liquid in a glass vial/ampoule and also supplied as a freeze-dried powder with 1 ml of water for injection I.P. for reconstitution. Each ml contains: enzyme refined, equine tetanus antitoxic immunoglobulin fragments not less than 1500 IU, cresol BP < 0.25% v/v as a preservative, glycine as a stabilizer BP, sodium Chloride BP, and water for Injections BP.

The Ethiopian Food Medicine and Healthcare Administration and Control Authority (FM-HACA), the former national regulatory body of Ethiopia licensed an equine tetanus antitoxin in 2015. Equine tetanus anti-toxin is manufactured by the ViNS Bioproducts Limited, India (Code: 130202084, A.W.No: 15/AAW/PI/02.00, DT: 25.04.2016) for marketing and it is in use in Ethiopia as post-exposure prophylaxis (PEP). The Pharmaceuticals Fund and Supply Agency (PFSA) of Ethiopia has included the product in the 2018 national pharmaceuticals procurement list (Serial No. 318, Base Code: Teta-30, Description: Tetanus Antitoxin (TAT), Equine - 1500 IU/ml in 1 ml Ampoule – Injection, Unite: 20) [28]. FM HACA renamed currently “Ethiopian Food and Drug Authority (EFDA)” procures and distributes the product to public healthcare facilities for prophylactic use against tetanus.

The product is delivered with the dose of 1000/1500 IU intramuscularly or subcutaneously to individuals at risk of tetanus infection for prophylactic purposes. The dose may be doubled or tripled in case of multiple and severe wounds. The prophylactic dose is also given in surgical operations as post-operative care. Before receiving the product, the individual needs to be tested for hypersensitivity, which is carried out by injecting 0.1 ml tetanus antitoxin serum in 1:10 dilution either subcutaneously or intradermally and observing for any local or general reaction for half an hour.

For children and adults, it is administered in 1500 IU single dose or 3000 IU if more than 24 h has elapsed [13]. It is administered following Besredka's method: inject 0.1 ml subcutaneously and wait for 15 min; if no local or general allergic reactions occur, inject 0.25 ml subcutaneously and wait for 15 min; if no reactions, administer the injection by IM route [13].

Injection of the antitoxin to individuals with a history of allergic reactions to equine protein and infantile eczema is contraindicated. As TAT is an animal source, it is expected to be antigenic, and hence type I hypersensitivity reaction which could be manifested as skin rashes and others is expected. If patients have eczema, TAT might exacerbate the condition. It is therefore contraindicated in such cases. The manufacturer advises that adrenaline injection (1:1000) must be available for immediate treatment of shock if it develops [27].

### Study participants, outcome measure and data analysis

The source population was all adults of at least 18 years of age and of either sex who visited the healthcare facilities seeking medical service for their tetanus-prone wounds. The study participants were all adults with tetanus-prone wounds who received the ViNS Bioproducts Limited’s equine TAT in the facilities for prophylactic use. Medical records of patients who visited the facilities, mainly at emergency, outpatient and surgery departments, were pooled from card rooms of each facility for the period from January 2015 to December 2019 when the TAT product of ViNS Biomedical Limited was in use. Medical records of eligible patients with tetanus-prone wounds who received the equine TAT were reviewed retrospectively for any adverse events following immunization according to the WHO definition for adverse events following immunization (AEFI) and serious AEFI [29]. The decision for inclusion as AEFI or Serious AEFI was based on the data recorded on patient cards by the physicians at the time of treatment. The magnitude and nature of the wounds, overall health status and demographic characteristics of the participants, and previous tetanus vaccination history of the participants were reviewed from the charts, 5-year registration books, and annual reports of the healthcare facilities and captured using a data collection tool adapted from the WHO AEFI guideline.

The outcome variable was AEFI and the independent variables were demographic variables (age, sex, residence and occupation), health status (general health condition, weight and height), nature of injury (place of injury, activity during injury, mechanism of injury, nature of injury, site of injury and time of injury) and vaccination history.

The primary outcome of the study was serious AEFI (Number of patients with serious AEFI arising from the TAT product of ViNS Bioproducts Limited) defined as the proportion of participants experiencing immediate AEFI related to the TAT, occurring within 2 h of administration of the TAT, measured as observed by the clinician or reported by the patient to the clinician, where the AEFI is explained by either of these conditions: (i) serum sickness, (ii) anaphylaxis (iii) seizures, (iv) abscess, (v) sepsis, (vi) encephalopathy, (vii) toxic shock syndrome, (ix) thrombocytopenia, (x) fever ≥ 38 °C.

In addition, the secondary outcome of the study was solicited AEFI (Number of Participants with solicited AEFI arising from the TAT product of ViNS Bioproducts Limited) defined as proportion of patients experiencing AEFI related to the TAT, commonly associated with local and systemic reactions, occurring greater than 2 h after administration, measured as observed by the clinician or reported by the patient to the clinician.

Data collected from each patient’s record were entered into EpiData software and exported to STAT version 14 for analysis. Baseline demographic characteristics of the patients were summarized descriptively using proportions and means. Baseline characteristics and nature of wounds were summarized in tables.

## Results

### Data characteristics

**Fig. 1:**
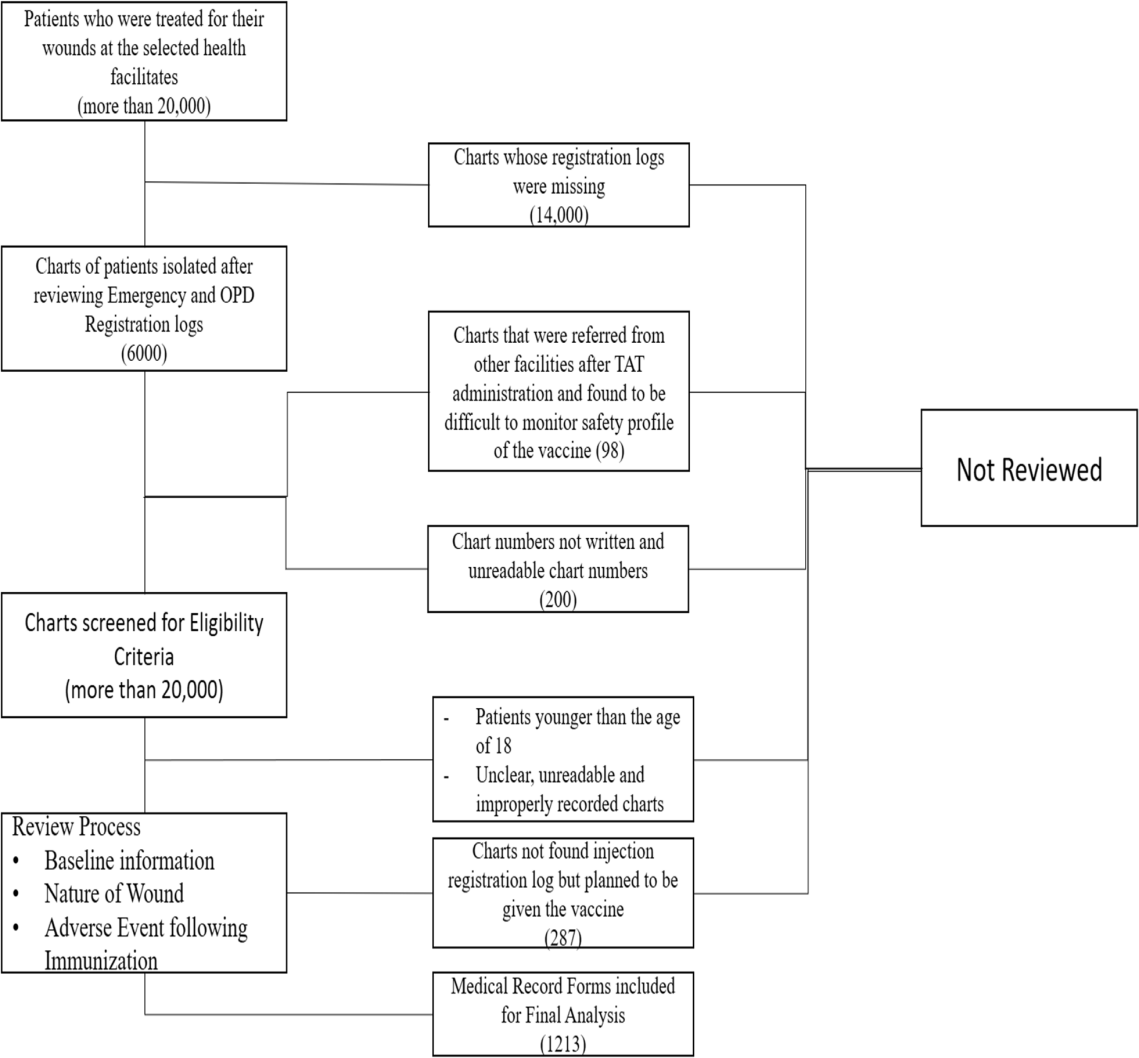
Flow charts on the identification of medical records eligible for review

There were more than 20,000 patients treated for trauma in the facilities from 2015 to 2019. Upon revision of available registration books, we identified 6000 charts to be eligible for the study, of which 1213 charts with complete and reliable data on the AEFI profile of the TAT were included in the final analysis. Figure 1 summarizes the flow and procedure followed in the identification of medical records eligible for review.

### Patients characteristics

**Fig. 2:**
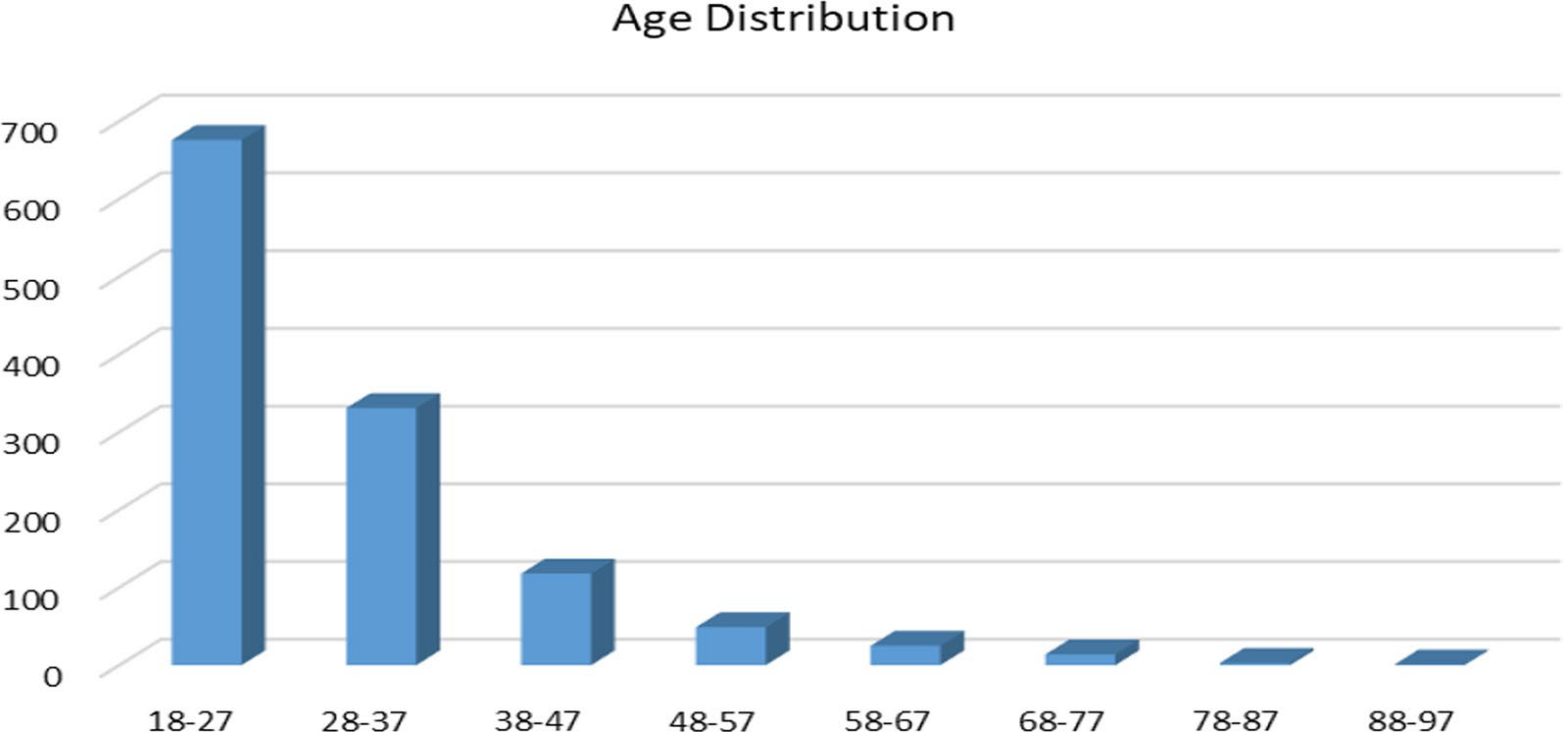
Age distribution of study participants

Table 1 summarizes the characteristics of patients included in the study. For the total of 1213 patients, the median age was 26 years (IQR = 11 years, and ranging 18–91 years) and 78% (949) were male with a male: female ratio of 3.6:1. The age distribution of the study participants is presented on Fig 2.

**Table 1:** Demographic characteristics of study participants

Characteristics	# Participants	%
Sex		
Male	949	78.23
Female	264	21.77
Residence		
Urban	1154	95.13
Rural	59	4.86
Literacy		
Literate	782	64.46
Illiterate	21	1.74
Unknown	410	33.80
Occupation		
Unemployed	10	0.82
Student	12	0.99
House wife	4	0.33
Trader	1	0.08
Farmer	17	1.40
Government employee	2	0.16
Daily laborer	4	0.33
Other	16	1.32
Unknown	1147	94.57
Marital status		
Never married	16	96.46
Married	25	2.06
Widower	2	0.16
Unknown	1170	96.46
Educational status		
None	15	1.24
Elementary	12	0.99
Secondary	12	0.99
Preparatory	7	0.58
University (diploma)	3	0.24
University (degree)	1	0.08
University (MSc., PhD)	1	0.08
Unknown	1162	95.80
TT vaccination history		
Yes	12	0.99
No	9	0.74
Unknown	1192	98.27

Most of the participants (*n* = 1154, 95.13%) were from urban areas and 64.46% were literate. The mode age lays with 18–27 years. TT vaccination status were recorded in the charts for 21 (1.73%) patients.

### Nature of wounds

The tetanus-prone wounds resulted mainly from stab (44%, 535) and blunt force (30%, 362), and the most common sites of wounds were hand (22%, 270) and head (21%, 253). The most and least frequently occurring types of wounds were open wounds (77%, 930) and organ system injury (0.003%, 4), respectively. Table 2 summarizes the nature and mechanism of occurrence of the wounds.

**Table 2:** Activities, nature and mechanism of wounds

Wound characteristics	# Participants	%
Place of injury		
Work	240	19.79
Education	2	0.16
Sport	26	2.14
Travelling	229	18.88
Unknown	386	31.82
Other	330	27.21
Nature		
Open wound	930	63.48
Fracture	107	7.30
Sprain/strain	25	1.71
Cut/bite	296	20.21
Bruise	103	7.03
Organ system injury	4	0.27
Mechanism		
Traffic	169	13.93
Assault	2	0.16
Fall	133	10.96
Blunt force	364	30.01
Stab/cut	536	44.20
Gunshot	7	0.58
Fire/heat	2	0.16

A person may experience more than one type of wound (nature)

The mean time of presenting at health facilities from the onset of trauma was 2.96 h, with the earliest and late time of presenting after 5 min and 30 days, respectively. Those people coming from school, working and suffered open wounds and traffic accidents were the ones who came earlier to hospitals. The modal time of presenting at health facilities was 15–30 min.

### Adverse event following immunization

Of the total 1231 patients, one severe AEFI and no solicited cases were observed. One 23-year-old male patient who presented within 3 h after experiencing a wound on his nose at the workplace had a severe local reaction immediately after injection of the TAT. He was given TAT 3000 IU intramuscularly soon after he was admitted. There were skin rash near the injection site. However, no other record was found on the medical record form. No AEFI was recorded for the other patients. Since the patients included in the study were adults 18 years of age and above, the majority (*n* = 1139, 93.89%) were given 3000 IU, while three patients received 1500 IU.

## Discussion

This study evaluated the safety of TAT developed and manufactured by the ViNS Bioproducts Limited, India, through reviewing medical records of patients with tetanus-prone wounds who received the TAT. The study reviewed medical records of 1231 eligible patients, of whom one male patient had a severe local reaction immediately after injection of the TAT, while no AEFI was recorded for the other patients. The patient who had AEFI was seen to have skin rash near the injection site. The documented tetanus-prone wounds resulted mainly from stab and blunt force, and the most common sites of wounds were hand and head.

Despite our findings, some studies conducted elsewhere reported AEFI of equine TAT, including anaphylaxis and late serum sickness [15, 23, 24, 30]; thus, we may have the risk of having undocumented adverse events reported in previous studies and other adverse events which were not previously reported. No death has been reported in our study. TAT products are in large use in many African countries as they are much cheaper than TIG [17, 29]. TIG persists for much longer in serum (4–5 weeks) than does TAT and seems to have fewer side reactions [14]. Due to its human origin, TIG can be applied directly without skin test, unlike TAT; however, since it is a human blood product, potential risk of infections with human viruses, such as viral hepatitis, HIV/AIDS, and other infectious diseases, still remains [25]. In the current time, equine TAT is in large use in Ethiopia and the product is administered to the patients in need both in public and private healthcare facilities.

In this study, 30% of the patients with tetanus-prone wounds received the TAT after 3 h, and there were one tetanus case in one of the study sites before the study period who developed the case without receiving the TAT vaccine. Though they came at a reasonable time, the main reason why it took them this much time was because the patients showed up in the healthcare facilities lately after taking some measures on their own or because they do not have nearby access to a healthcare facility. The health bureau in charge, with technical support from TAT suppliers, should devise mechanisms to educate the community on the urgency of seeking care in times of tetanus-prone wounds. According to the Ethiopian Federal Ministry of Health [FMoH] healthcare facilities’ capacity assessment held in 2016, 42% (1598/ 3804) of healthcare facilities lack TAT, though 89% of the facilities have tetanus toxoid vaccine [31]. The FMoH standard treatment guideline recommends the consideration of tetanus and rabies prophylaxis for all wounds [32].

The findings show that 24% of the patients with tetanus-prone wounds had been exposed to it at their workplaces, which is in agreement with a previous study done in Ethiopia that reported 39% of the exposer was at their workplaces [33]. Despite the need to fulfill workplace safety conditions in these settings, the workers need to have nearby access to TAT. Investigations of the current regulatory measures and what percent of workers in Ethiopia are protected in their workplace are needed.

Globally majority of tetanus cases are birth associated among new borns and mothers, Ethiopia is one of the 14 countries that has not reached the global maternal and neontal tetanus elimination goal. Even when it is known full immunization of pregnant women with tetanus injection reduces neonatal mortality by half [34–36]. TT immunization history is still low. In this study, only 0.13% of the patients had a recorded history of active immunization. A previous study conducted in Ethiopia reported that 70% of participants included in their study had no information about their active immunization history [19]; on contrary, 89% of the health facilities had access to TT [24]. These low level of immunization and lack of information about their active immunization history indicates a huge gap in knowledge and awareness about the vaccination and its benefit in the population. Studies carried out in the developed world reported 22–63.4% recorded history of active immunization [29].

The current study was hampered by the poor data recording system of the healthcare facilities under study and the retrospective study design employed that may hamper the level of evidence. On the contrary, the study was multicenter that captured data from a larger number of patients with tetanus-prone wounds in a resource-constrained country context where equine TAT is still in large use.

## Conclusion

The adverse event following immunization of the equine tetanus antitoxin produced by the ViNS Bioproducts Limited was vare rare. A regular review of the product’s safety performance and systematic collection and analysis of adverse event reports are important to ensure the safety of the product. A prospective study in real-life scenarios may supplement the current retrospective study that may have missed tracking untoward effects. The availability and safety of both active and passive immunization for tetanus including the prophylactic use of TAT for post-tetanus-prone wound needs to be widespread in respective communities.

## Data Availability

All relevant data are within the manuscript and its supporting information files.

## Abbreviations

AEFI: Adverse event following immunization
CDT-Africa: Center for Innovative Drug Development and Therapeutic Trials for Africa
EFDA: Ethiopian Food and Drug Authority
FMoH: Ministry of Health
HIV: Human Immunodeficiency Virus
IRB: Institutional Review Board
IU: International Unit
PEP: Post Exposure Prophylaxis
PFSA: Fund Supply Agency
TAT: Tetanus antitoxin
TIG: Tetanus immunoglobulin
TT: Tetanus toxoid
WHO: World Health Organization
